# Study of Mechanical and Sanitary Properties of Artificial Cast Stone Products

**DOI:** 10.3390/ma16031009

**Published:** 2023-01-21

**Authors:** Regita Bendikiene, Audrius Jutas, Paulius Nagys, Ausra Sipailiene

**Affiliations:** 1 Department of Production Engineering, Kaunas University of Technology, Studentu Str. 56, 51424 Kaunas, Lithuania; 2 Department of Mechanical Engineering, Kaunas University of Technology, Studentu Str. 56, 51424 Kaunas, Lithuania; 3 JSC Gforma, Drobės Str. 29G, 45189 Kaunas, Lithuania; 4 Department of Food Science and Technology, Kaunas University of Technology, Radvilėnų Str. 19, 50254 Kaunas, Lithuania

**Keywords:** artificial cast stone, thermoforming, bending test, flexural modulus, microbiological research

## Abstract

This paper aimed to analyse the mechanical and sanitary properties of artificial cast stone. To create an artificial stone product of the desired shape and design, a thermoforming process is used, during which heavy presses shape the artificial stone parts at a certain temperature. According to experimental studies, the most suitable temperature for monochromatic and patterned cast stone thermoforming is 160 °C when the material has the least bending resistance and does not react strongly to heat. It is observed that the cast stone bends more easily as the distance increases. The bending test of the glued samples made it possible to find out which of the three gluing methods is the most resistant to the forces acting on the samples during bending. The sanitary properties of the artificial stone were compared with the properties of other commonly used surfaces. In the microbiological study, suspensions of three different bacteria were spread on stainless steel, laminated particleboard and artificial cast stone, and washes were taken from 100 cm^2^ after 25 min. The results showed that the artificial stone after washing had the lowest number of colonies forming units per cm^2^, which supports the claim of excellent sanitary properties of the product.

## 1. Introduction

In light of current trends in the furniture and construction industry, manufacturers are striving to create a product that is as rigid and resistant to external factors as possible, and therefore opts for stronger and more dirt-resistant materials, especially when the product is used in public areas with special hygiene requirements. At the end of the 20th century, an acrylic mineral material was developed that met all of the above requirements [1]. Artificial stone is a modern and innovative solution for forming special-purpose surfaces, which today is one of the most popular alternatives to traditional surface materials. This material is used not only in the private sector but also in the public sector—from the countertop used in the domestic customer’s kitchen suite to the surfaces used in the airport reception and the premises of medical institutions. Some of the advantages of this material are its resistance to external factors, sterility and environmental friendliness. The service life of artificial cast stone products is long enough because of the technical possibilities to renovate and renew them, and the material is not only economical but also sustainable. At the same time, artificial cast stone is resistant to chemicals. It does not emit any gases at room temperature, as this material is made from 1/3 of polymethyl methacrylate (PMMA) and 2/3 of naturally occurring minerals that are not volatile or toxic to the environment [2]. Despite all the benefits of this material, manufacturers face the challenge of custom manufacturing. Artificial cast stone of different compositions, patterns or even colours reacts differently to the process of thermal moulding. Every cast stone product manufacturer needs to produce a product that meets the customer’s needs and solves all the subtleties of the created design. The user is informed of the limits of the mechanical properties of artificial cast stone and how those properties can be changed by changing the thickness of the countertop. This information is not relevant to the consumer, but the manufacturer needs to know the consumer has been informed and acquainted, thus relieving himself of some responsibility in the event of a product breakdown or accident. Beyond the mechanical properties of the artificial cast stone during the rampant COVID-19 pandemic, there was a greater need to disinfect surfaces to prevent the spread of the virus, not only in public treatment centres but even at home. The sanitary properties of surfaces have become another feature of the surface operation of domestic and public premises [3].

One can choose from a very wide variety of materials to make furniture and its components. Each of them has different performance characteristics, subtleties of the production process and service life. Some are chosen because of the attractive price-quality ratio, others because of the ability to provide sophisticated design solutions or aspects of luxury. In line with the principles of the circular economy and the trends of greater environmental awareness, the furniture and wood industry today seek to use environmentally friendly, durable, non-toxic materials [4], and the COVID-19 pandemic has also focused on sanitation. Although the aim is to follow sustainable production trends, most furniture and its components are still made of laminated particleboard (LMDP) and fibreboard (MDF) [3], which are attractive to consumers in terms of price and quality. The most expensive element in the construction of all cabinet furniture is the countertop or front facade. When choosing a desktop or countertop, priority is usually given to colour spectrum and patterns, design options, and structural strength and reliability [5]. Many of these listed properties are matched by artificial cast stone, which has occupied a significant place in the furniture manufacturing market for more than a decade. However, no matter how attractive and impeccable the products of this material look, the processes of artificial stone formation in production pose considerable challenges.

The composition of this material dictates the conditions of formation at certain temperatures (150 °C to 160 °C), and this property is exploited to give the products different shapes [6]. During the thermoforming process, it has been observed in production that the maximum bending angle (r) of artificial cast stone is highly dependent on the pattern or colour of the material. Each colour or pattern has been found to react differently to the thermoforming process due to the amount of minerals present in the material that does not become more flexible when exposed to temperature.

The minimalist design that became popular in the 21st century, generally classic in look, turned the design and production of furniture towards the emergence of single-colour modular furniture and thin countertops [7]. This design trend is also reflected in the market for artificial cast stone production, as more and more original 12 mm thick artificial stone products are being chosen. This choice is in line with current design trends, but to maintain a height of 12 mm, the worktop cannot be reinforced with additional wooden structures or thickened with an additional layer of artificial cast stone, and metal profiles make the product very expensive. For these reasons, the mechanical parameters of the 12 mm worktop are reduced. Moreover, during the COVID-19 pandemic, the requirements for countertops increased, and it became important that the surface be easy to clean and resistant to various chemical cleaners [3].

Artificial cast stone has been on the market for over a decade. Biochemist Donald Hillman Scolum is the inventor and pioneer of this material, working for EI Du Pont de Nemours and Co., now known as DuPont who first patented a purified polymethyl methacrylate product and process in 1968 (US3405088A) [8].

These past 53 years constitutes a relatively short period in the furniture industry. By contrast, natural wood and glass products have existed for many centuries during which these materials have changed their form and composition, wood-based materials have developed, and several scientific experimental studies have investigated the behaviour and properties of furniture materials. Such experiments are lacking in the field of artificial cast stone formation and exploitation: only a small number of international scientists have conducted related research in the last 53 years. Most research into artificial stone has been conducted on the use of it in the construction sector, the health sector and the aerospace industry. No such published research has been found in the furniture industry (for the production of countertops and sinks, bathroom components and cabinet furniture facades). Due to the growing demand for artificial cast stone products in Lithuania, there is therefore a need to investigate the performance and mechanical properties of this material [2].

The thermal formation of artificial cast stone has received little research. To-date, its study has focused more on the construction industry market and limited itself to a single colourmaterial, comparing materials developed by different artificial stone manufacturers [1,2].

Therefore, the issues addressed in this study differ from previous studies in that the aim will be to determine whether the minerals in the artificial cast stone affect the thermoforming process and to identify the appropriate temperature to form the artificial cast stone of different compositions. There are also no studies comparing the strength of countertops of different thicknesses. In the process of forming countertops, the choice of method of gluing artificial cast stone slabs is relevant, yet this is not specified in the works published by manufacturers and other scientists. Antibacterial surface properties are an important criterion when choosing a material for the production of a furniture panel, facade and countertop. With the onset of the COVID-19 pandemic, more attention was paid to surface properties. International researchers have found that the virus persists on surfaces for 3 to 16 h [3]. These figures have made not only furniture manufacturers but also consumers think about the anti-bacterial properties of furniture surfaces. Although many bacteriological studies of surfaces have been carried out in recent years, they are insufficient in view of the current situation which calls for more attention to be paid to the antibacterial properties of surfaces.

## 2. Materials and Methods

### 2.1. Materials

Two types of 12 cm thick cast stone slabs from DuPont Corian® were chosen for the study: these are the monochrome white Glacier White artificial stone (defined as A in this paper) and a patterned artificial stone rich in mineral pigments Smoke Drift Prima (defined as B). Artificial cast stone slabs are made of 2/3 aluminium (III) oxide and 1/3 polymethyl methacrylate (PMMA) [1]. The composition of the artificial stone is shown in Table 1.

PMMA is one of the polymers obtained by the polymerization of methyl methacrylate monomers. This material is commonly referred to as plexiglass [9], which acts as an adhesive in an artificial cast stone product [2]. Aluminium (III) oxide is a natural substance extracted from the mineral bauxite (aluminium ore). It is odourless, non-toxic, non-carcinogenic and resistant to corrosion; the alumina is naturally white and easily absorbs pigments of other colours; and this mineral performs the function of a filler in the composition of artificial cast stone. To give the material a different colour and pattern, the composition of the material is supplemented with different mineral substances—namely pigments [2]. The mechanical and physical properties of the artificial cast stone used in the test are given in Table 2 as provided by the manufacturer of this material.

### 2.2. Preparation of Materials for Testing

**Figure 1:**
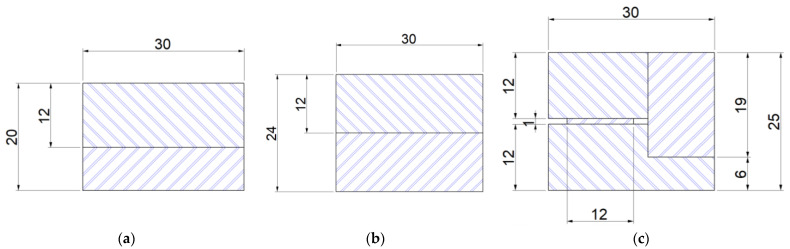
Cases of gluing of artificial cast stone: (**a**) H−20; (**b**) H−24; (**c**) H−25.

*Glacier White* (A) and *Smoke Drift Prima* (B) artificial cast stone samples (36 samples in total) with dimensions 120 mm × 30 mm × 12 mm were cut using a cutting machine Altendorf F45 for the bending at different temperatures. Eight specimens were prepared for the test of glued artificial cast stone structures produced from A samples. Dimensions of glued samples are provided in Table 3. Three different gluing methods (Figure 1) were used for gluing the artificial cast stone slabs.

**Figure 2:**
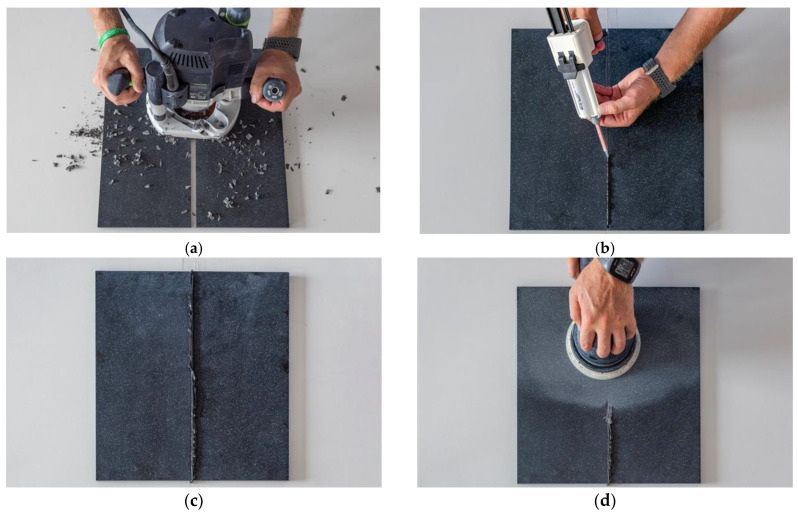
Stages in the process of gluing artificial cast stone: (**a**) levelling of the gluing area; (**b**) gluing; (**c**) drying the glue; (**d**) grinding and polishing.

The specimens were glued using the adhesive specified by the manufacturer and the technical recommendations for gluing artificial cast stone (Figure 2). Before the operation, the gluing area of the sample was slightly sanded to improve the adhesion of the artificial cast stone surfaces to the adhesive. The gluing area was then sterile wiped with an alcohol swab to remove all dirt, dust and grease from the gluing surface. After cleaning, the material was glued and compressed with clamps. When the glue dried, the samples were calibrated using a format cutting machine Altendorf F45 (Altendorf, Juszczyn, Poland).

### 2.3. Bending Test

A three-point bending method was used for the bending test, with one concentrated force (*F*) acting in the middle of the sample. Using this method, a net bending section is obtained in the middle of the sample, allowing the sample to break at the weakest point in that section.

The purpose of the bending test is to determine how the mechanical properties of the artificial cast stone change by bending it at different temperatures and using different gaps between the supports (*L* = 80 mm). The most effective method to compare the temperature at which a material bends best, and whether the distance between the supports affects the bending process, is to compare the modulus of elasticity of the material at different temperatures. The three-point bending method was also applied to bending tests with glued specimens; for this purpose, a gap of 100 mm between the supports (*L*) was selected only, and the specimens were not exposed to temperature during bending. To investigate which bonding method was the most resistant to bending, the bending normal stress σ at the breaking point of the specimens was chosen, with the formula [10]:

(1) $$\left|\sigma \right|=\left|\sigma_{z}\right|=\frac{FLy_{i}}{4I_{x}},$$

where *σ_z_* is the normal stress arising along the *z*-axis (Pa); *F* is the maximum force at fracture (N); *L* is the distance between supports (m); *y_i_* is the distance taken from the neutral *x*-axis (m); *I_x_* is the moment of inertia for a sample cross-section (m^4^)—which has expression *I_x_* = *BH*^3^/12, where *B* and *H* are the width (m) and the height (m) of rectangular shape, respectively; and *i* is the number of steps of integration. It should also be mentioned that the inertia moment equation for *I_x_*, which is presented above, is used for samples named H−12, H−20 and H−24, only; while for sample H−25, according to its geometric peculiarity, it may be expressed as follows *I_x_* = *I_x_*_1_ − *I_x_*_2_ = (*BH*^3^ − bh^3^)/12, where letters *b* and *h* satisfy the total width (m) and the height (m) of the two empty spaces, respectively, that are located at the level of the neutral *x*-axis and occupy a central position in terms of height *h*. At the section of fracture, a shear stress distribution has been calculated by the equation:

(2) $$\left|\tau \right|=\left|\tau_{zy}\right|=\left|\tau_{yz}\right|=\frac{FS_{xi}}{BI_{x}},$$

where *τ_zy_* is the shear stress varying along the *y*-axis and perpendicular to the *z*-axis (Pa); *S_xi_* is the one-half of the first cross-sectional moment (m^3^), which is expressed as *S_xi_* = *A_i_y_ci_*, where *A_i_* and *y_ci_* are the specified part of cross-sectional area (m^2^) and its centroid distance taken from *x*-axis (m), respectively. The modulus of elasticity of sample groups A and B during bending was calculated using the following formula [10]:

(3) $$E=\frac{FL^{3}}{48I_{x}d},$$

where *E* is the modulus of elasticity (Pa), and *d* is the *y*-axis displacement (m) corresponding to the point of force *F* in the experimental graph *F* = f(*d*). The test schedule for bending the artificial cast stone at different temperatures was as follows:—a total of 5 temperatures were selected for the study: 140 °C, 145 °C, 150 °C, 160 °C, and 170 °C; at each of the indicated temperatures, four samples of materials A and B were bent separately using different distances between the supports; the distances between the supports were 80 and 100 mm; bending speed was 5 mm/min; radius (*R*) of supports and bending device was 5 mm; the selected maximum deformation size was 16 mm; and before the bending test, the samples were stored at the intended temperature for 6 min. The test schedule for bending glued artificial stone was as follows—a total of 8 specimens were used for the study, for 4 different sizes and 3 gluing types: H−12 (non-glued), H−20, H−24 and H−25—the numbers indicating their thickness in mm; samples were labelled by height 5); 100 mm distance between supports was selected; and bending speed was 5 mm/min. During both tests, the samples were bent one by one using a universal test machine Tinius Olsen H10KT with a maximum permissible load of 10 kN, which has an integrated conventional heating chamber that allows tests at a constant temperature. During bending, the force (N) and displacement (mm) were obtained using the Qmat software of the universal test machine 5.37. After the tests, the results were processed and analysed. Before the bending test, the specimens were stored at room temperature and accurately measured with a calibrated calliper. It is worth noting that moisture has little effect on artificial stone because, as mentioned above, the material is non-porous due to its composition.

### 2.4. Microbiological Test

Four types of plates and three types of bacteria were used in the microbiological study: two artificial stone slabs, laminated particle board, and a stainless-steel sheet; the cultures of bacterial suspensions used for bacteriological examination were *Staphylococcus aureus* ATCC25923, *Salmonella typhymurium* ATCC14028, *Escherichia coli* ATCC25922 (Jong, S. C. ATCC Names of Industrial Fungi. Rockville, Md.: American Type Culture Collection, 1994).

Laminated particle board (LPB) is a material used in the furniture industry to make furniture or as an interior decoration element. LPB is made from recycled wood waste, and the boards are given different surface colours when using laminate. When pressing the particle board at a high temperature, both sides of the board are glued with laminate—paper soaked in melamine resin. The laminated surface is resistant to moisture and high temperatures, and is sufficiently hard. Laminates can be smooth or rough, often carved with various textures, imitating natural wood. Monochrome laminates can be used, which can be matt or glossy [11]. The panels produced by PFLEIDERER, which has been focused on producing laminated particle boards for many years and ensuring the highest standards, were used for the experiment (Table 4).

Stainless steel is a high-quality structural material that is mechanically strong and rigid and has high corrosion resistance due to its high chromium content. These properties determine the price of the material, making stainless steel one of the most expensive alloys on the market [12]. This study used AISI 304 (X5CrNi18-10) stainless steel, which is a popular grade of stainless steel (Table 5, [13]). This steel is often used in the chemical, automotive and food industries, in the production of professional and household kitchen equipment, and in electrical equipment. Although this class of steel is widely studied, it is still of interest to scientists: several studies have been conducted on its weldability, its ability to combine with other metal alloys and its resistance to aggressive environments [14].

The bacterial strains of *Salmonella typhimurium* (ATCC14028), Staphylococcus aureus (ATCC25923), and *Escherichia coli* (ATCC25922) were used for the antimicrobial assay. Bacterial strains were pre-cultivated overnight (18–20 h) on a slant plate count agar (Plate count agar, Liofilchelm) at 37 °C. After pre-cultivation, bacteria strains were individually suspended in sterile saline (0.85% NaCl) to obtain the inoculum equivalent to 0.5 McFarland standard.

A volume of 1 mL of the prepared suspension was individually spread evenly over a 200 cm^2^ area. Contaminated surfaces were left for 25 min for the bacteria to attach to the surface. After 25 min, the 100 cm^2^ area was washed with a sterile cotton swab. The remaining 100 cm^2^ was washed with a sterile damp cloth and again washed with a sterile cotton swab to take the attached bacteria from the surface. The cotton swab was washed in sterile saline (0.85% NaCl) and made into serial dilution that was spread on Petri plates with the appropriate medium: *Staphylococcus aureus*—Baird-Parker Agar Base medium, *Salmonella typhymurium*—XLD medium, and *Escherichia coli*—Eosin methylene base agar medium. The inoculated plates were incubated at 37 °C for 24 h (*Staphylococcus aureus* and *Salmonella typhymurium*) and 72 h (*Escherichia coli*).

*Staphylococcus aureus* (Golden Staphylococcus) is an opportunistic pathogen that causes a large proportion of infections in public places and hospitals. One of the reasons this bacterium is so prevalent is its pathogenic cells, which adhere strongly to a variety of surfaces and materials including natural and abiotic substances. As a result of its exceptional adhesion, *Staphylococcus aureus* bacteria deposited on the surface can continue to multiply and form due to its mechanically and chemically strong biofilm. Because of this film, the bacterium spreads rapidly in public places: clinics, buses, train stations and even at home, making the spread of this bacterium a big challenge for the health system, as well as cabinet furniture and material manufacturers [15].

*Salmonella typhymurium* (Salmonella) is one of the main bacteria that cause food poisoning in humans and animals. Salmonella is most often contracted by consuming raw and unprocessed products: eggs, meat, vegetables and even fruits. This bacterium can also spread on surfaces, but due to poor protection against external factors, it does not survive for long and cannot reproduce. The effects of salmonella are very harmful, e.g., in China alone, more than 80% of food poisoning is caused by this bacterium [16].

*Escherichia coli* (*E. coli*) is another bacterium that most commonly spreads through food and surfaces with food debris. These bacteria are usually harmless and rarely cause disease in a healthy person. However, they can be dangerous for children and those with a weakened immune system. This pathogen does not have good adhesion to surfaces, but under certain conditions, e.g., trapped in food residues in various surface cracks, this pathogen can persist for a considerable period, thus endangering the environment [17].

## 3. Results and Discussion

### 3.1. Bending Test at Elevated Temperatures

**Figure 3:**
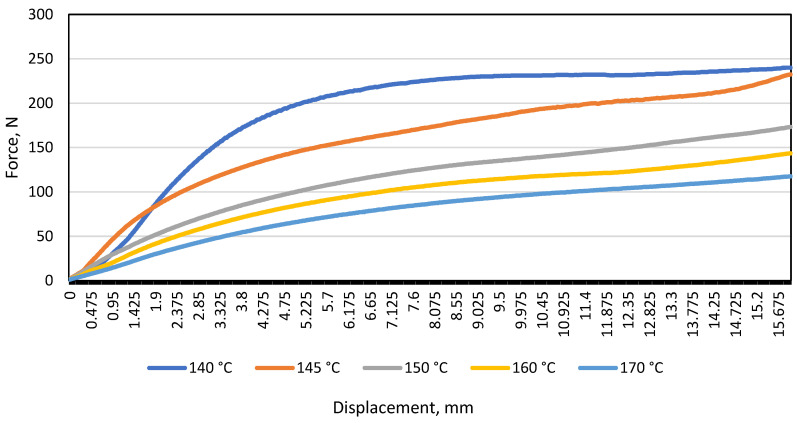
Load and displacement diagram of monochrome material A under bending at different temperatures when the distance between the supports was 80 mm.

The bending test was performed using a distance of 80 mm between the supports (*L*), on bending samples types A and B at all specified temperatures: 140 °C, 145 °C, 150 °C, 160 °C, and 170 °C. From the obtained data, averages were derived and a graph of deformation curves was plotted. It is seen in Figure 3 that the resistance of the material type A under load (N) decreases with increasing test temperature.

During the study, the maximum displacement (16 mm) was chosen by estimating that when the material displacement reaches the limit of ≈18 mm–20 mm, the material rests on the selected support edges, thus reducing the actual distance between the supports and distorting the resulting load and displacement data in the diagram.

**Figure 4:**
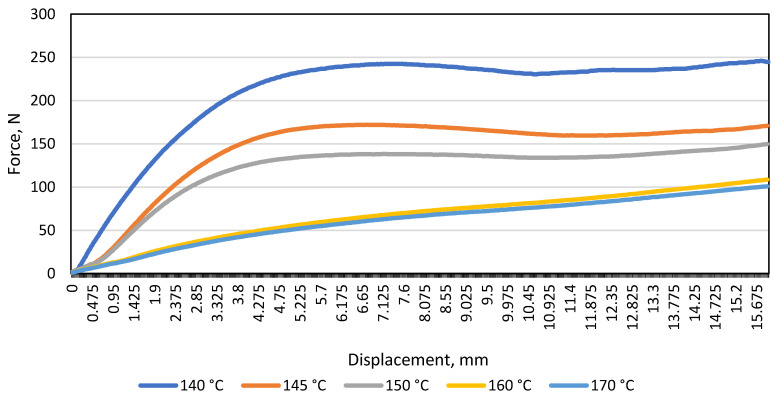
Diagram of the load and displacement of the material B during bending at different temperatures when the distance between the supports was 80 mm.

After testing the monochrome material A, a curve of the average load and displacement of the patterned material B was drawn at a distance between the supports of 80 mm (Figure 4).

**Figure 5:**
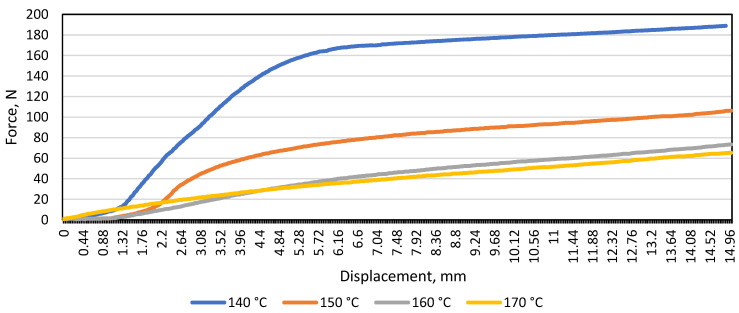
Diagram of load and displacement of monochrome material A under bending at different temperatures when the distance between the supports was 100 mm.

After examining the patterned material and plotting the average load and displacement diagram, the same trend was observed when testing the monochrome material: the resistance of the material to the load (N) decreases with increasing temperature. In the graph shown (Figure 4), it was observed that at 140 °C, 145 °C, and 150 °C, the patterned material bends further than the monochrome one (the maximum difference is 24 N). To find out whether the gap between the supports affects the modulus of elasticity (E), a diagram of the average load and displacement was drawn for the patterned and monochrome material, when the distance between the supports was 100 mm (Figure 5).

**Figure 6:**
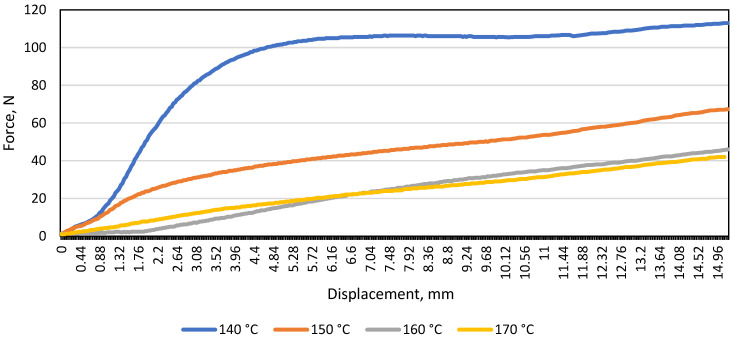
Diagram of the load and displacement of the patterned material B during bending at different temperatures when the distance between the supports was 100 mm.

Tests of monochrome artificial cast stone have shown that the resistance of the material to bending forces decreases with increasing material length. The temperature of 145 °C was not used in the tests when the distance between the supports was 100 mm; and a large difference in force and displacement is visible comparing samples bent at 140 °C and 150 °C. As in the case of monochrome sample tests, a large difference in force and displacement between 140 °C and 150 °C is observed at a distance of 100 mm between the supports (Figure 6).

Comparing Figure 5 and Figure 6, it was observed that when the distance between the supports was 80 mm, the monochrome material was significantly more resistant to bending than the patterned one. This means that changing the distance between the bending machine supports affected the bending resistance of the patterned material. It was decided to compare the modulus of elasticity at all temperatures and compare the patterned artificial stone with the monochrome.

**Figure 7:**
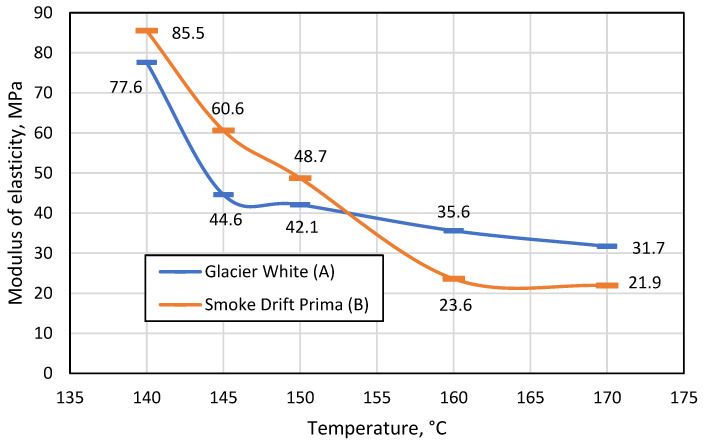
Change in modulus of elasticity (MPa) of monochrome A and patterned B materials with changing temperature.

In Figure 7, one observes that as the process temperature increases, the modulus of elasticity of the material decreases, which means that the material becomes less and less resistant to the bending force acting on it. The figure also shows that up to 150 °C, the monochrome artificial stone was more flexible than the patterned artificial stone, but at the temperature specified by the manufacturer (160 °C) and the higher temperature of 170 °C, the patterned material yields to the bending process more easily. The difference in modulus of elasticity when bending the patterned material at 140 °C, 145 °C, and 150 °C is on average 18.7 MPa, and at 160 °C and 170 °C only 4.1 MPa. Therefore, bending the patterned material in the temperature range of 140–150 °C is not recommended. By contrast, this cannot be stated when evaluating the results of the monochrome material; the modulus of elasticity differs by 33 MPa at 140 °C and 145 °C, and by 6.5 MPa at 150 °C and 160 °C. It follows that a monochrome material can also be thermoformed at 150 °C if it is not possible to reach 160 °C.

**Figure 8:**
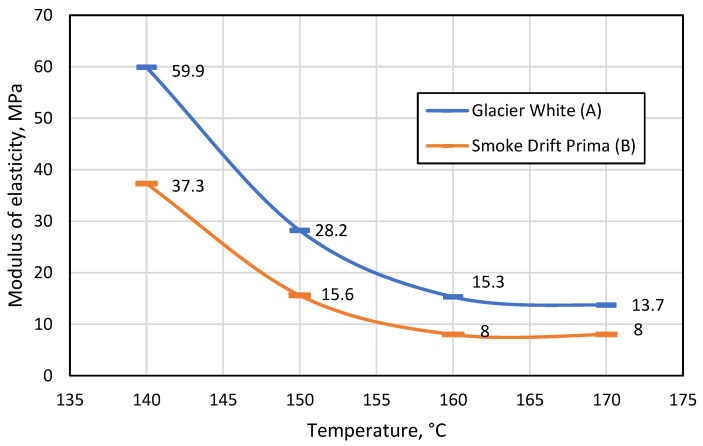
Change in the modulus of elasticity (MPa) of monochrome A and patterned B materials with increasing temperature when the distance between supports was 100 mm.

The test results (Figure 8) showed that when the distance between the supports was 100 mm, the monochrome material was almost 50% more resistant to the force applied during bending at all temperatures.

**Figure 9:**
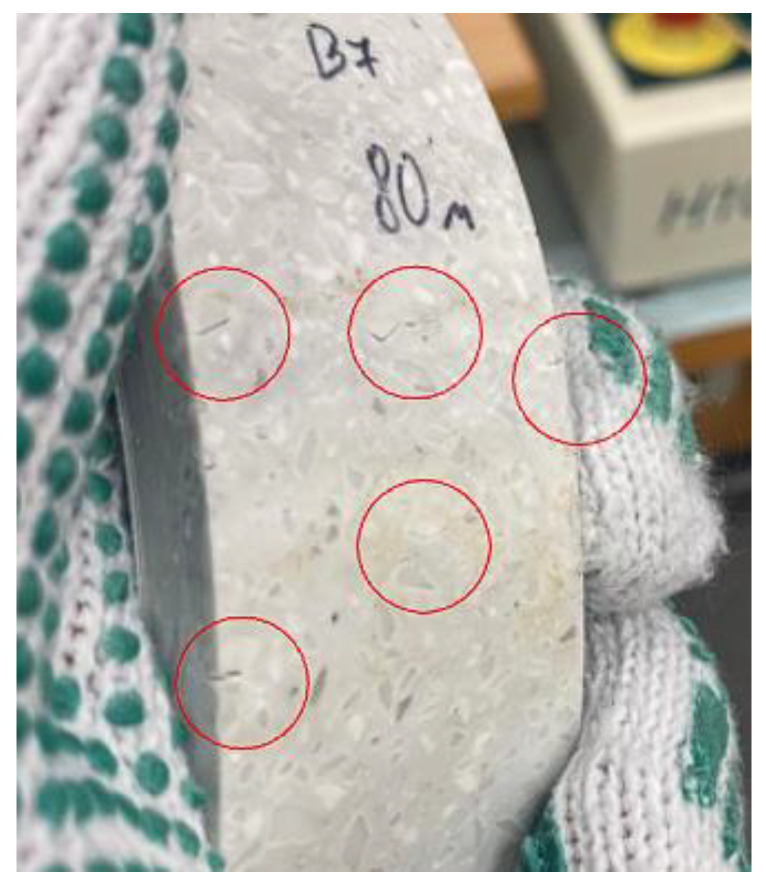
Patterned artificial cast stone after bending test at 170 °C (cracks are marked in red).

As the distance between the supports increases, the flexural strength of the patterned material decreases more than that of monochrome. It was also observed that the distance between the supports had a significant effect on the modulus of elasticity, and both materials were much more elastic when the gap between the bend supports was 100 mm compared to 80 mm. This suggests that the bending length should be considered during thermal forming. A comparison of the two figures shows that the most suitable temperature for thermal formation is in the range between 160 °C and 170 °C, as at these temperatures the difference between the modulus of elasticity is the smallest. However, at 170°C, it was observed that both monochrome and patterned artificial stone products begin to emit a sharp odour, begin to smoke slightly, and minimally change colour from lighter to darker. In addition, the patterned artificial stone begins to crack at the points between polymethyl methacrylate (PMMA) and minerals (Figure 9).

### 3.2. Bending Test of Glued Artificial Stone at Room Temperature

**Figure 10:**
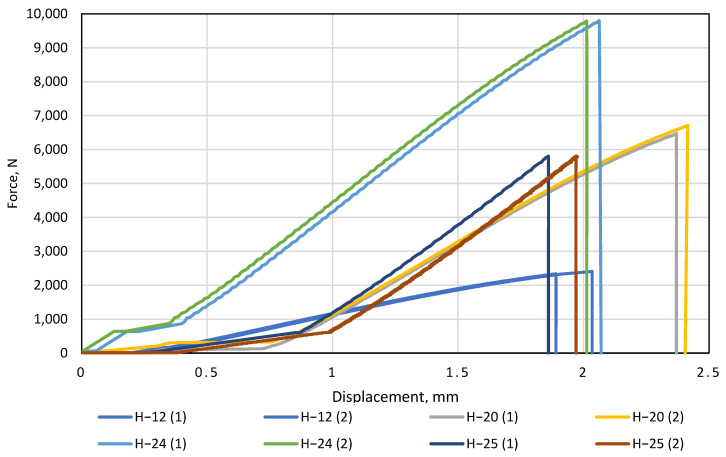
Load and displacement curves of glued specimens.

Tests of glued artificial stone were started by examining the non-glued specimens with the smallest thickness (12 mm) and ending with the largest (25 mm). From the obtained data, a graph of deformation curves was plotted, in which the force (N) was plotted on the *y*-axis and the displacement (mm) on the *x*-axis. The glued samples were compared to a standard 12 mm thick sample. In every case, two samples of the same gluing method were tested. We observe that the ascending trends of both curves of the non-glued sample H−12 are very similar (Figure 10). The difference between H−12(1) and H−12(2) is negligible, with the difference in displacement and force at the breaking point was 0.14 mm and 71 N, respectively. Figure 10 can be compared with Figure 3 and Figure 5, revealing a large difference in force and displacement when bending a 12 mm artificial stone when it is not exposed to temperature. This means that from 140 °C, the artificial stone achieves a much larger displacement and requires only a force of up to 300 N compared to a standard thickness of 12 mm artificial stone bent at room temperature.

Tests with a standard thickness of artificial stone were followed by a test with a glued artificial stone with a combined thickness of 20 mm. This sample height was achieved by gluing two artificial stone blanks on top of each other, one of which was glued to the original 12 mm and the other was levelled to 8 mm using a CNC machine (Figure 1a). It was observed that increasing the height of the specimen also increases its resistance to the force acting on it during bending. It can be seen that the displacement of the part at the breaking point remained very similar to that of the 12 mm samples (difference of only 0.3 mm), but the force at the breaking point was 2.7 times greater.

**Figure 11:**
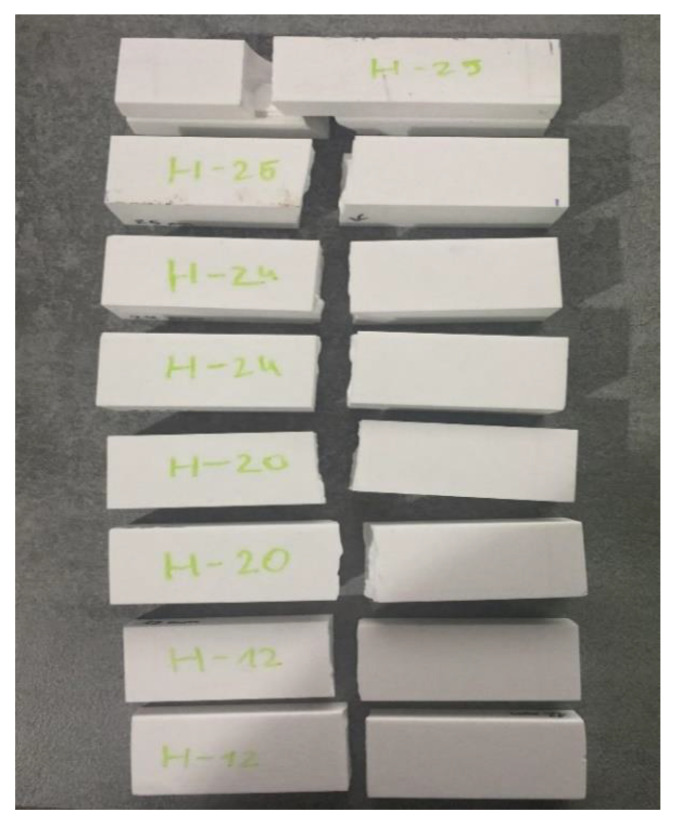
Glued artificial stone samples after bending test.

According to the results obtained, this gluing method is much more effective in terms of strength, but it needs to be emphasized that twice as much material was used for this sample. This was followed by samples with a height of 24 mm. This height was achieved by gluing two artificial stone blanks of equal size together (Figure 1b). This method of providing the height of the worktop is the most popular in production, as it does not require much preparation or additional milling before gluing. Figure 10 illustrates that the 24 mm thick artificial stone is more resistant to bending compared to the 12 mm and 24 mm thick samples. Both H−24 samples broke at an average load of 9790 N, close to the allowable load of Tinius Olsen H10KT, which is 10 KN. Comparing the H−24 gluing method with H−20, achieving 24 mm thicknesses in production is much easier and cheaper. The last glued thickness tested was 25 mm. This method of gluing is more unique complex to the gluing methods mentioned above because in its construction, one component stands vertically in the section (Figure 1c). It is obvious (Figure 10) that of all the gluing methods, the H−25 gluing method had the lowest resistance to the force applied during bending. This was because the structure had a part standing vertically, which reduced the possibility of the glued element achieving greater displacement during the bending test. For this reason, the sample had nowhere to expand during bending and broke faster than samples H−24 and H−20. During the study, all tests were performed without any problems or additional interference, and all bent parts were broken into two separate parts (Figure 11).

**Figure 12:**
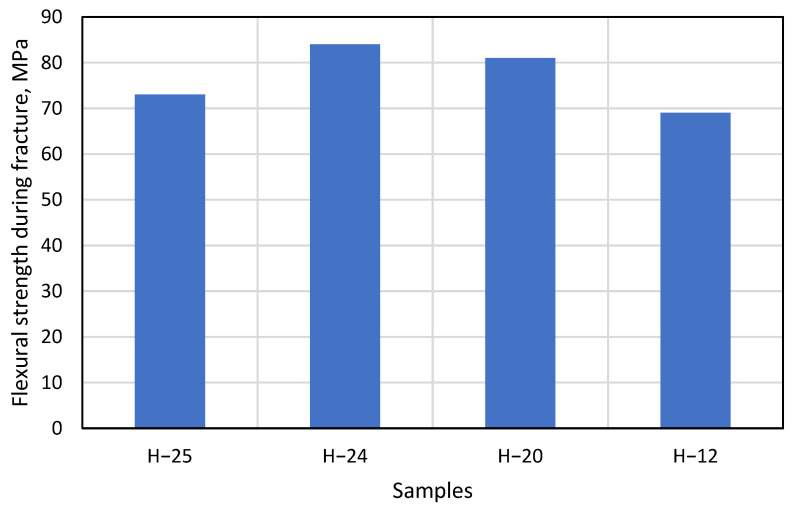
Change in flexural strength as the thickness of the material changes.

Under the right bending conditions and exposed to temperatures, artificial stone breaks elastically, very similarly to plastic—even leaving white marks at the fracture site. However, in this study, the artificial stone was not exposed to temperature, resulting in specimens breaking as standard brittle materials. The last step of this experiment is to calculate and compare the mean flexural strength of the samples H−12, H−20, H−24, and H−25 during fracture (Figure 12).

Data in Figure 12 shows that the H−25 gluing method has the lowest flexural strength value compared to other gluing methods. This is due to the design solutions of the product. The assembly of this gluing method contains a vertical part at the bending point, which prevents the product from bending and causes the specimen to break faster. Thus, the gluing area of this sample is the smallest compared to other samples. In the experimental studies, it was observed that the H−24 and H−20 samples had a high resistance to bending, which is reflected in the graph of the change in flexural strength: the flexural strength of H−24 at break was 84 MPa compared to 81 MPa for H−20. Comparing the results of these two gluing methods, it is observed that the difference between them is only 3 MPa, which confirms the fact that the constructions of both H−24 and H−20 samples are related and that both have a similar flexural strength during fracture.

Based on the results of the studies and analysis, ones can conclude that the H−24 and H−20 samples have the highest resistance to bending force. Despite the complex construction and the height of the H−25 samples, the gluing method employed caused the samples to break during the bending test under lower loads and to have a lower average tensile strength (by 67 MPa). It was also observed that the flexural strength of H−12 corresponded to the flexural strength of 68.95 MPa as indicated by the manufacturer in Figure 12.

### 3.3. Stress State Evaluation of Glued Specimens

To show the general impact of both normal *σ* and shear *τ* stresses, they were combined in the von Mises equation for a case of general plane stress, into which the results obtained from Equations (1) and (2) had been put. Given the duality of shear stresses in an isotropic continuous medium, and assuming that the entire stress tensor can be expressed as:

(4) $$\bar{\sigma }={\left\{\sigma_{x},\sigma_{y},\sigma_{z},\tau_{xy},\tau_{yz},\tau_{zx}\right\}}^{T}={\left\{0,0,\sigma_{z},0,\tau_{yz},0\right\}}^{T},$$

then the final von Mises equation, which takes only the active stresses *σ_z_* and *τ_zy_*, is simplified as follows:

(5) $$\sigma_{v}=\sqrt{\sigma_{z}^{2}+3\tau_{zy}^{2}}=\sqrt{\sigma^{2}+3\tau^{2}}.$$

**Figure 13:**
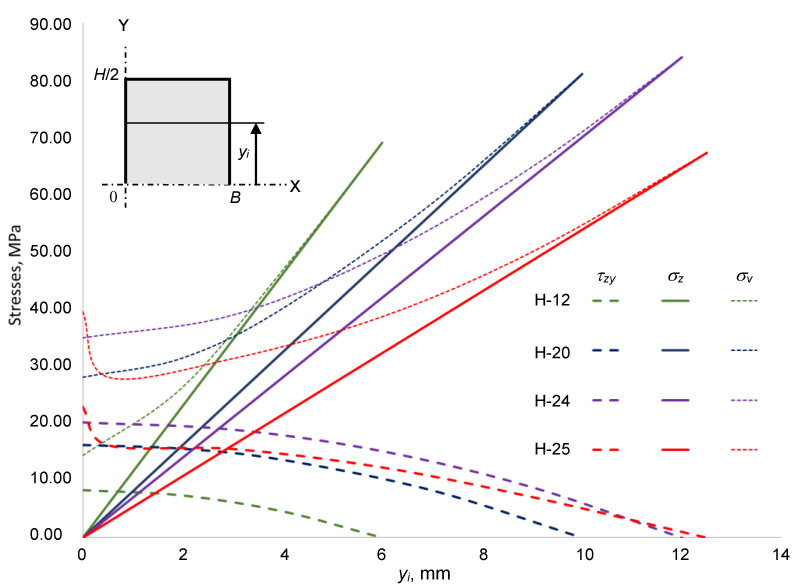
Distribution of stresses at different cross-sectional levels taken along the *y*-axis.

The graph below shows the change in operating stresses concerning the increasing value of distance *y_i_* on half of the cross-sectional area of each specimen (see Figure 13).

It is observed that the combined normal *σ_z_* and shear *τ_zy_* stresses determine the specificity of von Mises stress *σ_v_*. Sample H−25 should be explained here, as the gluing scheme results in a threshold of the tangential stress *τ_zy_* curve at the gap location (the gap ends at 0.5 mm), whereas normal stress *σ_z_* has no significant effect because the gap is close to the neutral line where the latter stress is 0.

At the sample cross-sectional centroid, von Mises’ stress *σ_v_* starts at a higher value than shear stress *τ* by the multiplier $\sqrt{3\;}\left(\sigma_{v}=\sqrt{3}\tau \right)$. This phenomenon is the same for all the samples. However, in sample H−25, where the level of *y_i_* = 0.5 mm, a sudden drop is seen which shows a significant influence of the gap; this forms a concave shape with the gradual increase in von Mises stress *σ_v_* along the changing distance *y_i_* until the maximum of the normal stress *σ_z_* is reached, at which point *σ_z_* = *σ_v_*.

This bonding scheme of the sample H−25 and the phenomenon of shear stress *τ* distribution affect the earlier delamination along the *z*-axis which results in the earlier fracture reaching a much lower level of the deformation force *F* (see Figure 10). This delamination has a special character, the so-called *fracture steps* where the two fracture areas of glued components are shifted from each other—as presented in Figure 11. The described technological properties play an important role in quantifying the assembly and gluing of components with or without gaps.

As the main aim of the mechanical investigation was the stress state, the principal stresses *σ*_1_, *σ*_2_ and maximal shear stress *τ*_max_ were used.

**Figure 14:**
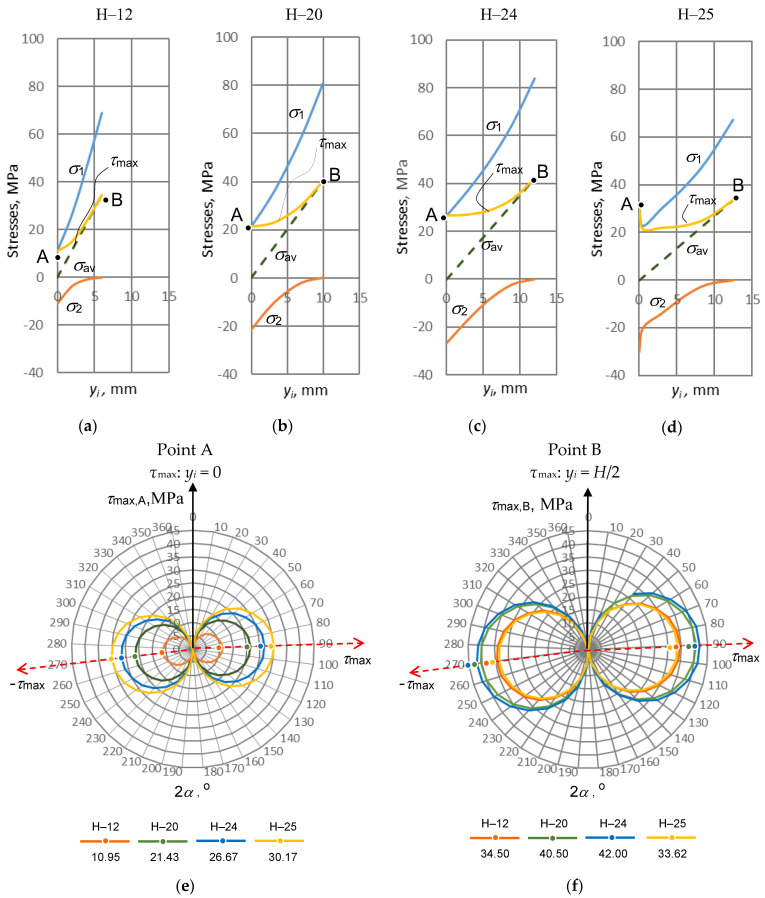
The representation of stresses *σ*_1_, *σ*_2_, *τ*_max_ and *σ*_av_ versus variable cross-sectional distance *y_i_*: (**a**–**d**) a set of stresses acting within the samples H−12, H−20, H−24, H−25, respectively; (**e**,**f**) the maximal shear stresses at point A (*τ*_max,A_) and Point B (*τ*_max,B_), respectively. The two red dashed arrows indicate the directions where the shear stresses reach extreme values $\left|\tau_{\max}\right|$.

These stresses reveal information about the extreme stress values and their location. In addition, the authors calculated the average principal stress *σ*_av_, which illustratively showed a distribution connecting all the aforementioned stresses. As is usual in this field comparative analysis of stresses, we took the standard approach to calculate principal stresses in terms of Mohr’s circle, which graphically illustrates a stress state at a single cross-sectional point that might be located by any distance *y_i_* taken from the neutral *x*-axis, as mentioned in the discussion about Equation (1). In this work, the authors decided not to plot numerous Mohr’s circles at every integrating step changing by *y_i_* for all the series of samples because of the inconvenience and unnecessary efforts for a reader to readily compare them. All graphs were plotted on two axes, which are the stresses *σ* and the variable distance *y_i_*, respectively (see Figure 14). All the stresses, which are mentioned in this paragraph, are presented below as follows:

− the principal stresses

(6) $$\sigma_{1,2}=\frac{\sigma_{z}}{2}\pm \sqrt{\frac{\sigma_{z}^{2}}{4}+\tau_{zy}^{2}},$$

where σ_1_ > 0 and σ_2_ ≤ 0;

− the maximal shear stresses

(7) $$\tau_{\max}=\frac{\sigma_{\max}-\sigma_{\min}}{2}=\frac{\sigma_{1}-\sigma_{2}}{2},$$

− the maximal shear stresses

(8) $$\sigma_{\mathrm{av}}=\frac{\sigma_{\max}+\sigma_{\min}}{2}=\frac{\sigma_{1}+\sigma_{2}}{2}.$$

Results obtained using Equations (6) and (4) would have a structure such as:

(9) $$\bar{\sigma }={\left\{\sigma_{1},\sigma_{2},0\right\}}^{T}.$$

As follows from the above equations, the state of stresses at any point is divided into four quantitative stress criteria that vary along the *y*-axis: *σ*_1_, *σ*_2_, *τ*_max_ and *σ*_av_.

The point A was taken as a reference located at the neutral *x*-axis (cross-sectional centroid, *y_i_* = 0 mm) while point B takes a distance lifted along the same *y*-axis up to the specimen’s outer surface, where *y_i_* = *H*/2. To reasonably interpret the calculation results, all graphs were plotted using the same scales for variable cross-section height *y_i_* (scale is 0–15 mm) and for the four stresses (scale is −40–100 MPa). All the calculation results are presented in Figure 14.

In all the samples, the principal stress *σ*_1_ starts acting at point A when *y_i_* = 0 mm. As it is seen from Equations (1), (2) and (6), the stresses are *σ*_z_ = 0, *τ*_zy_ ≠ 0 and *σ*_1_ = *τ*_zy_ ≠ 0, respectively, which means the leading stress, responsible for the stress state, is the shear stress *τ*_zy_. At Point B (*y_i_* = *H*/2), the identical stresses are *σ*_z_ ≠ 0, *τ*_zy_ = 0 and *σ*_1_ = *σ*_z_ ≠ 0, and the leading stress is now *σ*_z_.

Continuing this interpretation with the principal stress *σ*_2_, which also starts at the same point A as stress *σ*_1_, we may observe a situation when *σ*_2_ < 0. Not only do we have a negative stress *σ*_2_, but we also see a symmetry of absolute principal stresses, that is, |*σ*_1_| = |*σ*_2_|.

This fact takes stresses *σ*_z_ = 0, *τ*_zy_ ≠ 0 and a negative value *σ*_2_ which is influenced by the structure of Equation (6) and regarded with so called *mirror symmetry* of maximal shear stress *τ*_max_ < 0 in Mohr’s circle, if the angle in the latter is α = 3π/4 degrees (sin2α < 0). Here, the stress *τ*_zy_ is also the leading one. At Point B, the stresses are the following: *σ*_z_ ≠ 0, *τ*_zy_ = 0 and *σ*_2_ = *τ*_zy_ = 0. Here, the leading stress is also *σ*_z_.

The maximal shear stress *τ*_max_ acts in all the *y_i_* range 0 ≤ *y_i_* ≤ *H*/2, where at the distance *y_i_* = 0, the first represents point A; and at *y_i_* = *H*/2, it represents point B. In all the graphs pictured in Figure 14a–d, we can see that *τ*_max,A,B_ > 0 and *τ*_max,A_ < *τ*_max,B_ because under bending conditions, the following tendency exists: *τ*_zy,A_ < *σ*_z,B_ (Figure 13).

The average of the principle stresses *σ*_av_ takes a half of the sum of both principle stresses *σ*_1_ and *σ*_2,_ and always retains their algebraic symmetry in all the *y_i_* range 0 ≤ *y_i_* ≤ *H*/2 (from point A to Point B) as follows: *σ*_av,A_ = *σ*_1_ − *σ*_z_/2 and *σ*_av,B_ = *σ*_2_ + *σ*_z_/2. The stress *σ*_av_ varies in the range 0 ≤ *σ*_av_ ≤ *σ*_z_/2.

After reviewing the changes in stresses *σ*_1_, *σ*_2_, *τ*_max_ and *σ*_av_, the main trends can be summarized. At point A, the stresses *σ*_1_ and *σ*_2_ are obtained because they are influenced by the stress *τ*_zy_, which is decisive at the centre when *σ*_z_ = 0. At point B, the identical stresses *σ*_1_ and *σ*_2_ depend on the normal stress *σ*_z_ because at the surface, they are critical when *τ*_zy_ = 0. It should be also said that point A joins the beginning of both principal stress *σ*_1_ and maximal shear stress *τ*_max_, whereas point B combines the highest level of maximal shear stress *τ*_max_ and the highest end point of the average principal stress *σ*_av_, when *τ*_max,A_ = *σ*_1,A_ and *τ*_max,B_ = *σ*_av,B_, respectively.

The above discussed generalizations could be presented in the following form:

(10) $$\sigma \left(0\leq y_{i}\leq \frac{H}{2}\right)=\{\begin{array}{c} \;\sigma_{1}>0:\;\;\;\;\;\;\;\;\;\tau_{zy}\leq \sigma_{1}\leq \sigma_{z};\\ \sigma_{2}\leq 0:\;\;\;\;-\tau_{zy}\leq \sigma_{2}\leq 0;\\ \;\;\tau_{\max}>0:\;\;\;\;\;\;\;\;\;\tau_{zy}\leq \tau_{\max}\leq \frac{\sigma_{z}}{2};\\ \;\;\;\sigma_{\mathrm{av}}\leq 0:\;\;\;\;\;\;\;\;\;\;\;\;\;0\leq \sigma_{\mathrm{av}}\leq \frac{\sigma_{z}}{2}. \end{array}.$$

The results of the stress analysis, generalized by Equation (10), helped the authors to distinguish the importance of cross-sectional peculiarities and to make some important remarks. For this reason, the change in maximal shear stresses *τ*_max_ have been taken in accordance with the cross-sectional geometry of tested samples as presented in Figure 14d,e, in which the stress values are given below the name of the sample. The maximal shear stresses *τ*_max_ was split into the two extreme values corresponding to the centroid of cross-section (Point A) and the outer surface (Point B), respectively. The two sine functions were used for their transformation in the form −*τ*_max_ ≤ *τ*_zy_ (sin2α ≤ *τ*_max_) for point A, and −*τ*_max_ ≤ *σ*_z_/2(sin2 α ≤*τ*_max_) for point B. In more detail, the above transformations may be presented as follows:

− at point A

(11) $$\tau_{\max}{\left(\alpha \right)}_{y_{i}=0}=\{\begin{array}{c} \tau_{\mathrm{zy}}\sin2\alpha \geq 0,\;\mathrm{if}\;0\leq \alpha \leq \frac{\pi }{2}\;;\;\;\\ \tau_{\mathrm{zy}}\sin2\alpha \leq 0,\;\mathrm{if}\frac{\pi }{2}\leq \alpha \leq \pi \;;\\ \tau_{\max}=\tau_{\max}\left(\frac{\pi }{4}\right),-\tau_{\max}=\tau_{\max}\left(\frac{3\pi }{4}\right). \end{array}$$

− at point B

(12)  τmax(α)yi=H2={σz2sin2α≥0, if 0≤α≤π2 ; σz2sin2α≤0, ifπ2≤α≤π ;τmax=τmax(π4),−τmax=τmax(3π4).

The sample H–25 (the outside yellow circle) showed the worst technological result under bending because the maximal shear stress *τ*_max_ is the highest at point A, that is *τ*_max_ = 30.17 MPa, whereas at point B, the identical stress values are located on the internal circle where *τ*_max_ = 33.62 MPa. This fact may also explain the delamination of the glued specimen with certain geometric cross-sectional features of sample H–25.

Moreover, as seen in Figure 14a–d, the geometry of cross-section influences how shear stress at point A is far from that at point B. Clearly, with larger *H*/2, the stresses *τ*_max_ for the same points tend to come much closer. The closer they are, the higher their ratio *τ*_max,A_/*τ*_max,B_. The above ratio illustrates the appropriate sensitivity to geometry, and this can be generalized as follows:

(13) ns(τ)=|τmax|yi=0|τmax|yi=H2≤1

Due to the changing height of the cross-section and the accompanying bending stiffness *EI_x_*, the difference between the maximum tangential stresses *τ*_max_ in the centre and the surface decrease, while their ratio approaches to 1. For the samples H–12, H–20, H–24, and H–25, the ratios *n_s_*_(*τ*)_ are 0.317, 0.529, 0.635, and 0.897, respectively. This ratio may be relevant when delamination is significant and the maximal stress *τ*_max_ on the neutral axis is located outside (Sample H–25) as shown in Figure 14e. Therefore, it can be used as a quantitative criterion for testing substances that tend to delaminate or weaken, as discussed in this work. The ratio can also be applied to testing for defects that cause premature breakage of specimens when tested under bending conditions.

### 3.4. Microbiological Examination

When choosing surface materials in the furniture and construction industry, the highest requirements are placed on the mechanical properties, price and durability of the material. However, the current situation in the world has forced manufacturers to pay increasing attention to the sanitary properties of the material. With the outbreak of the COVID-19 pandemic, there has been a great need to disinfect surfaces, not only in health care, and public sector businesses, but even at home, to prevent the virus from spreading. The World Health Organization states that the COVID-19 virus stays in the air for 3 to 16 h and settles on surfaces for an average of 3 to 4 days. Therefore, surface cleaning and disinfection have become a very important procedure to ensure eliminating the virus. Without cleaning countertops and other surfaces, viruses and bacteria start to multiply after 20 min, not only on the horizontal surfaces of countertops but also in joints between countertop panels, and between washbasin and countertop [3].

The most popular surface materials are laminated particle board (LMDP), stainless steel or natural wood. However, LMDP surfaces are often coated with acrylonitrile-butadiene-styrene (ABS) and glued together with polyurethane adhesive (PUR). Such a combination of materials does not guarantee effective sanitary properties: often, water penetrates through the gluing seam and begins the process of swelling the board. As a result of such damage to the LMDP surface, fungus and bacteria grow in the damaged medium. Natural wood or stone, on the other hand, does not face this problem, yet these materials are porous and require sealing to prevent bacteria from multiplying within the media. The stone and wood industries have been developing antibacterial sealants for many years, but it is very difficult to ensure that the surface manufacturer has impregnated each product properly. It should also be noted that a sink often needs to be installed within a countertop. Sinks are often installed with putty or other silicone sealants whose sanitary effectiveness is limited.

One of the materials on the market that does not face the problems mentioned here is artificial cast stone. The surface of this product, made of aluminium and acrylic polymers, has a special strength, is odourless, dust-free, and homogeneous, and does not require a base for fixing. The surfaces of the cast stone are combined into a homogeneous product, so there is no favourable place for viruses, bacteria and microorganisms to survive and multiply. Due to its structure and properties, non-porous cast stone has antibacterial properties and is widely used in laboratories, medical institutions, catering establishments, and the food industry, where it is necessary to ensure the sterility of surfaces and stop the spread of the current COVID-19 virus. Such material ensures greater sterility even when the surface has not been completely disinfected. As artificial stone adhesives are made of the same material as the material itself, they do not require silicone or acrylic sealants.

**Figure 15:**
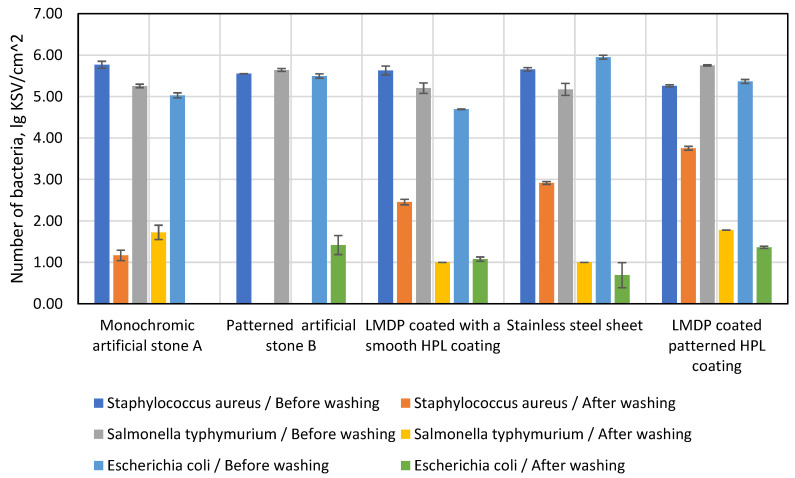
The number of bacteria on the surface of each material.

Therefore, the purpose of this study was to investigate the sanitary properties of monochrome and patterned artificial cast stone, two different laminated particle boards and stainless steel. The logarithmic mean of the bacterial counts on the surface of each material before and after washing is shown in Figure 15.

Patterned and monochrome artificial stone had 5.77/5.55 lg CFU/cm^2^ (number of colonies forming units per square centimetre) before washing, and after washing this number dropped to 1.17/0 lg CFU/cm^2^. It was observed that no bacteria per square centimetre were present in the artificially cast monochrome stone after washing. The test of artificial cast stone was followed by a test of LMDP coated with a smooth HPL coating, which had 5.63 lg CFU/cm^2^ before and 2.46 lg CFU/cm^2^ after washing. In a stainless-steel plate experiment, it was observed that the *Staphylococcus aureus* bacteria survived well after washing (2.92 lg CFU/cm^2^), although it had fewer bacteria than the working pre-wash stone (5.66 lg CFU/cm^2^). The worst results in the study with this bacterium were shown by the LMDP-coated corrugated HPL plate, which had 5.26 lg CFU/cm^2^ before washing and 3.75 lg CFU/cm^2^ after washing.

A second bacterial strain used for the study was *Salmonella typhymurium*. As in the study with the *Staphylococcus aureus* colony, a patterned artificial stone showing no bacteria after washing showed the best results. The LMDP was coated with a smooth HPL coating and the stainless-steel plate contained about 5.30 lg CFU/cm^2^ before washing and about 1.73 lg CFU/cm^2^ after washing. Monochrome artificial stone and LMDP with corrugated HPL coating contained ~5.4 lg CFU/cm^2^ before washing and 1.75 lg CFU/cm^2^ remained after washing: they were the most effective against this bacterium.

The last bacterial strain used in this study was *Escherichia coli*. This test showed the best sanitary properties to belong to a monochrome artificial stone that contained 5.03 lg CFU/cm^2^ before washing with none remaining on the cleaned surface after washing. The sanitary properties of the monochrome artificial cast stone did not significantly underperform the stainless-steel plate, which contained 5.95 lg CFU/cm^2^ before washing, with only 0.69 lg CFU/cm^2^ remaining after washing. In contrast to the bacterial colonies used before, the patterned artificial cast stone, which contained 5.49 lg CFU/cm^2^ before washing and 1.42 lg CFU/cm^2^ after washing, showed poorer sanitary properties. Both LMDPs showed approximately the same results—5.0 lg CFU/cm^2^ before washing and 1.22 lg CFU/cm^2^ after washing.

## 4. Conclusions

1. For both the patterned and monochrome varieties, artificial cast stone has the lowest values of modulus of elasticity in the temperature range 160–170 °C (on average 22 MPa at a distance between supports of 80 mm, and 8 MPa for 100 mm). This means that at these temperatures, the material is least resistant to bending forces. It is recommended to avoid temperatures of 170 °C or higher, as the substance reacts noticeably to temperature: it changes colour, emits a pungent odour and smokes.

2. By changing the distance between the supports during bending, it was found that the working length of the bent part affects the modulus of elasticity. The values of the modulus of elasticity were 3 times higher in the temperatures range of 160–170 °C, when the distance between the supports was 80 mm. Therefore, we conclude that when manufacturing sinks, countertops and other products from artificial cast stone using bending forms, it is necessary to pay attention to the working length of the bent part.

3. The bending tests of glued artificial cast stone showed that the H–20 and H–24 samples had the highest resistance to bending forces, with an average flexural strength of 83 MPa at the time of sample fracture for H–20, and only 73 MPa for H–25. The results obtained suggest that the H–20 and H–24 methods should be chosen for the production of mechanically strong artificial cast stone products.

4. The fracture occurs in the outer tensile layers at the maximal von Mises stress *σ_v_* in samples H–12 (not glued), H–20 and H–24 without observable delamination of the glued samples. Since the inner layers of the samples remain undamaged, von Mises stress *σ_v_* increases their dimensions: 69 MPa, 81 MPa, and 84 MPa, respectively. The cause of fracture is the maximal normal stress *σ*_z_.

5. The cross-sectional features of sample H–25 influence the lowest von Mises stress, *σ_v_* = 67.2 MPa. The maximal shear stress *τ*_max_ acts in the middle of all the samples; however, the beginning of delamination was observed only in the sample H–25 at maximal shear stress *τ*_max_ equal 30.2 MPa, while in samples H–12, H–20 and H–24, the same stresses are significantly lower: 10.9 MPa, 21.4 MPa and 26.7 MPa, respectively. Despite the larger dimensions of the H–25 sample, its maximal shear stress *τ*_max_ causes delamination and earlier fracture.

6. The best sanitary properties were exhibited by artificial cast stone, which had 2 times fewer colony-forming units per square centimetre (0.79 lg CFU/cm^2^) than stainless steel (1.54 lg CFU/cm^2^) and 3 times less than LMDP after cleaning the bacterial surface (1.9 lg CFU/cm^2^).

## Figures and Tables

**Table 1:** Composition of artificial stone.

Ingredient	CAS Number	Percent, %
Aluminium trihydroxide	216645-51-2	55–66
Polymethyl methacrylate	9011-14-7	34–45
Iron oxide	12227-89-3	3
Mesoporous carbon	1333-86-4	3
Titanium dioxide	13463-67-7	3
Methyl methacrylate	80-62-6	1
Dyes	–	1

**Table 2:** Mechanical and physical properties as provided by the artificial stone manufacturer.

Physical Properties		
Properties	Standard	Values
Density	ASTM D792	1700 kg/cm^3^
Thermal expansion	ASTM E228	3.9 ×10^−5^ m/m °C
**Mechanical Properties**		
Bending strength	ASTM D790	68.95 MPa
Compressive strength	ASTM C365	110.32 MPa
Rockwell hardness	ASM D785	>85

**Table 3:** Characteristics of glued artificial stone.

Sample	Colour	Type	Number	Length	Width	Height
				mm		
H−12 *	*Glacier White*	Monochromic	2	120	30	12
H−20			2			20
H−24			2			24
H−25			2			25

* not glued.

**Table 4:** The physical, chemical and mechanical properties of the LPB as provided by the manufacturer.

Physical Properties	
Properties	Values
Density (EN 323:1993)	750 kg/m^3^
Humidity (EN 317:1993)	5–7%
**Chemical Properties**	
Toxicity	DYN 120:E-1
**Mechanical Properties**	
Elastic modulus when bending longitudinally (EN 310:1993)	3400 N/mm^2^
Modulus of elasticity when bending transversely (EN 310:1993)	1400 N/mm^2^

**Table 5:** Chemical composition of steel X5CrNi18-10 according to EN 10088-2-2005.

Steel	Chemical Composition, wt. %							
X5CrNi18-10	C	Si	Mn	Ni	P	S	Cr	N
	0.7	≥1	≥2	8–10.5	≥0.045	≥0.015	17.5–19.5	≥0.11

## Data Availability

Not applicable.
